# Motor learning of mice lacking cerebellar Purkinje cells

**DOI:** 10.3389/fnana.2013.00004

**Published:** 2013-04-23

**Authors:** M. Elena Porras-García, Rocío Ruiz, Eva M. Pérez-Villegas, José Á. Armengol

**Affiliations:** ^1^División de Neurociencias, Departamento de Fisiología, Anatomía y Biología Celular, Área de Anatomía y Embriología Humana y Fisiología, Universidad Pablo de OlavideSeville, Spain; ^2^y Fisiología, Universidad Pablo de OlavideSeville, Spain

**Keywords:** cerebellum, motor learning, *Lurcher*, *tambaleante*, Purkinje cells

## Abstract

The cerebellum plays a key role in the acquisition and execution of motor tasks whose physiological foundations were postulated on Purkinje cells' long-term depression (LTD). Numerous research efforts have been focused on understanding the cerebellum as a site of learning and/or memory storage. However, the controversy on which part of the cerebellum participates in motor learning, and how the process takes place, remains unsolved. In fact, it has been suggested that cerebellar cortex, deep cerebellar nuclei, and/or their combination with some brain structures other than the cerebellum are responsible for motor learning. Different experimental approaches have been used to tackle this question (cerebellar lesions, pharmacological agonist and/or antagonist of cerebellar neurotransmitters, virus tract tracings, etc.). One of these approaches is the study of spontaneous mutations affecting the cerebellar cortex and depriving it of its main input–output organizer (i.e., the Purkinje cell). In this review, we discuss the results obtained in our laboratory in motor learning of both *Lurcher* (Lc/+) and *tambaleante* (*tbl/tbl*) mice as models of Purkinje-cell-devoid cerebellum.

## Introduction

The cerebellum coordinates motor activities to be performed or already underway. In fact, cerebellar damage produces disturbance in movements and in body support. The relationship between cerebellum and motor learning was first suggested with the studies of Ramón y Cajal (1911), Dow and Moruzzi (1958), and Eccles et al. (1967). Dow and Moruzzi (1958) hypothesized that the cerebellum contributes to motor learning by determining how to perform accurate and correct movements. Thereafter, numerous studies have been devoted to analyzing the role of the cerebellum in perceptive and cognitive processes. Thus, the essential contribution of Marr, localizing the site of motor learning in the cerebellar cortex (Marr, 1969), and the later application of Marr's theory to the classical conditioning (Albus, 1971), whose physiological basis are directly related to *long-term depression* (LTD) mechanisms (Ito, 1989), defined the neuronal circuit involved in associative motor learning which remains accepted and discussed.

The anatomically highly refined organization of the cerebellum and its afferent/efferent pattern of projections from/to motor and premotor regions of cerebral cortex and spinal cord provides a paradigmatic substrate supporting its participation in motor behavior and learning (see Bernard et al., 2012). Furthermore, the analysis of the development of the cerebellar hemispheres and the expansion of the cerebral cortex in phylogeny also suggests the involvement of the cerebellum in cognitive functions (Leiner et al., 1986). The topography of the cerebellar cortex is closely related to deep cerebellar nuclei arrangement which also have different motor function according to the region of the cerebellum in which they are situated, and each nucleus controls a different aspect of the movement for the whole-body map (Thach et al., 1992; Thach, 1997).

Numerous studies have been performed in order to determine the role of the different cerebellar parts as a site of motor learning and/or memory storage. Among the authors who give a central role to the cerebellum, some point to the cerebellar cortex (Attwell et al., 2001; Chen et al., 1996), some to the deep cerebellar nuclei (Clark et al., 1992; Bracha et al., 2001), while others postulate the coordinated work of cerebellar and extra-cerebellar regions (Aou et al., 1992; Delgado-García and Gruart, 2002; Christian and Thompson, 2003; Koekkoek et al., 2003; Jiménez-Díaz et al., 2004; Porras-García et al., 2005, 2010; Sánchez-Campusano et al., 2007, 2009; Freeman and Steinmetz, 2011). Different experimental approaches, such as retrograde trace with virus (Morcuende et al., 2002), lesions, or pharmacological studies of cerebellar structures (Yeo et al., 1985a,b; Bracha et al., 1999; Christian and Thompson, 2003; Jiménez-Díaz et al., 2004), electrophysiological recordings from cerebellar cortex and nuclear neurons (Gruart et al., 2000; Porras-García et al., 2010), the study of cerebellar developmental disorders (Manto and Jissendi, 2012), and the use of mutant mice (Chen et al., 1996; Grüsser-Cornehls and Bäurle, 2001; Koekkoek et al., 2003, 2005; Porras-García et al., 2005, 2010), have been used to elucidate the function of the cerebellum. For this last approach, some mutations resulting in a total loss of Purkinje cells are very useful. Here, we summarize the results obtained in *tambaleante* and *Lurcher* mutant mice as models used for this purpose.

## *Lurcher* mutation

The *Lurcher* mutation appeared spontaneously in 1954 in the mouse colony of the Medical Research Council Radiobiological Research Unit at Harwell, England. In 1960, Phillips described motor-coordination problems associated to the *Lurcher* mutation. He also reported that this mutation was semi-dominant and the gene was localized on chromosome 6 (Phillips, 1960). The *Lurcher* mutation is caused by mutation in the δ2 glutamate receptor (GluRδ2; Caddy and Biscoe, 1979; Zuo et al., 1997). GluRδ2 is predominantly expressed in both Purkinje cells and several hindbrain cells (Araki et al., 1993; Lomeli et al., 1993; Mayat et al., 1995; Takayama et al., 1995, 1996; Landsend et al., 1997). Homozygous *Lurcher* mice (*Lc/Lc*) die after birth (P0) through a massive loss of mid- and hind-brain cells (Cheng and Heintz, 1997; Resibois et al., 1997). In contrast, the heterozygous *Lurcher* mouse (*Lc*/+) suffers cerebellar Purkinje cell death from the third and fourth day after birth (P3–P4) (Swisher and Wilson, 1977). From the 8th day after birth (P8), the Purkinje cell loss produces the degeneration of granule cells and olivary neurons (Caddy and Biscoe, 1979). Three months after birth, the *Lc*/+ mouse has lost almost every Purkinje cell, some 90% of granule cells, and some 75% of the olivary neurons (Caddy and Biscoe, 1979; Wetts and Herrup, 1982; Heckroth and Eisenman, 1991; Norman et al., 1995; Wullner et al., 1995; Doughty et al., 2000).

**Figure 1:**
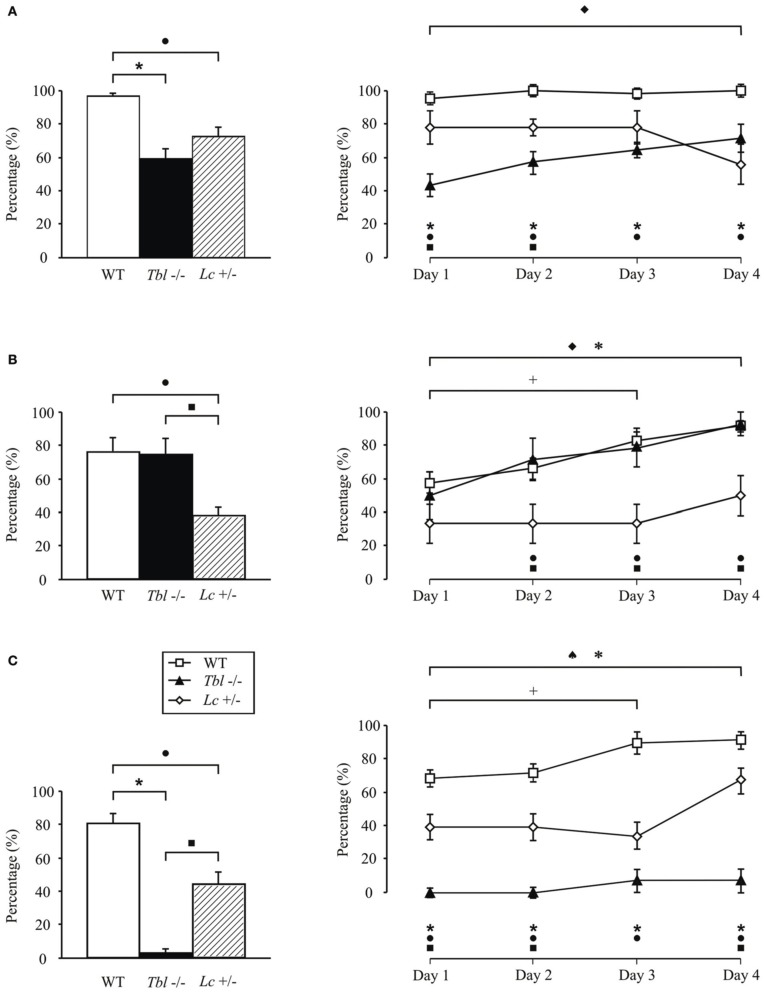
**Comparison of motor activity and motor learning in *tambaleante* (*tbl*^−/−^), Lurcher (*Lc*/+), and wild-type (WT) mice evaluated in various motor tasks: fall (A), horizontal bar (B), and vertical pole (C) performed twice a day during 4 days.** Significant differences were found between groups in the percentage (%) mean values (left) [One-Way ANOVA *F*-test, *F*_(6, 1158)_ = 17.50 (fall); *F*_(6, 1158)_ = 8.45 (horizontal bar); *F*_(6, 1158)_ = 45.28 (vertical pole), *P* < 0.05] as well as in the temporal evolution for each of the tests (right). [Two-Way ANOVA *F*-test, *F*_(4, 360)_ = 2.41 (fall); *F*_(4, 360)_ = 0.56 (horizontal bar); *F*_(4, 360)_ = 1.62 (vertical pole), *P* < 0.05]. ♦, significant differences between *tambaleante* mice; +, between different sessions of wild-type animals; ♠, between *Lurcher* mice; *, between *tambaleante* and wild-type mice; ■, between *tambaleante* and *Lurcher* animals; and •, between *Lurcher* and wild-type mice. *Lurcher* mice data collected from Porras-García et al. (2005).

Despite the motor problems that appeared in *Lc*/+ mice in various motor tests performed (fall, rotarod, ladder, horizontal bar, eyeblink classical conditioning), they were able to learn new motor tasks, but the amplitude of the learned responses were significantly lower than in wild-type mice (Lalonde, 1994; Caston et al., 1995; Le Marec et al., 1997; Hilber and Caston, 2001; Porras-García et al., 2005) (Figure 1). However, the lesion of the interpositus nucleus prevented the generation of conditioned eyeblink responses in *Lc*/+ and wild-type mice (Yeo et al., 1985a; Welsh and Harvey, 1989; Bracha et al., 1999; Jiménez-Díaz et al., 2004; Porras-García et al., 2010). Moreover, electrophysiological recordings of interpositus and red nuclei in *Lurcher* mice during the eyeblink classical conditioning suggest compensatory mechanisms in the absence of cerebellar cortex during performance of learned movements (Porras-García et al., 2010). These results suggest that deep cerebellar nuclear neurons (interpositus and dentate nuclei) may be involved more in the modulation and proper performance of ongoing conditioned responses than in their generation and/or initiation during learning processes (Gruart et al., 1997; Delgado-García and Gruart, 2002; Jiménez-Díaz et al., 2004).

## *Tambaleante* mutation

**Figure 2:**
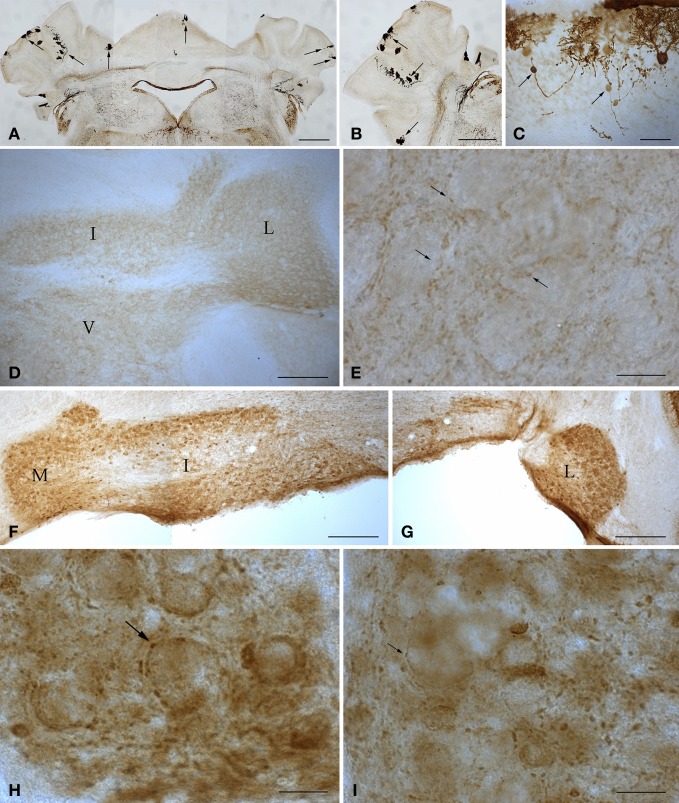
**Microphotographs of coronal (A, D–I) and sagittal (B,C) sections immunostained with anti-calbindin (A–C) and anti-parvalbumin (D–I) antibodies illustrate the main features of the cerebellum of wild type and 1-year-old *tambaleante* mice.** Scarce Purkinje cells remain throughout the cortex (**A–B**, arrows), preserving a mirror location on both sides of the cerebellar cortex (**A**, arrows). Axons of degenerating Purkinje cells show typical axonal torpedoes (**C**, arrows). Parvalbumin immunoreactivity is restricted to small endings (**E**, arrows), and is absent in both deep cerebellar and vestibular nuclei neuronal somata **(D)**, in wild-type cerebellum. In *tambaleante* cerebellum, parvalbumin immunoreactivity is present in neuronal somata of deep cerebellar nuclei **(F,G)**, and in large (**H**, arrow) and small (**I**, arrow) terminal endings. I, L, and M, interposed, lateral, and medial cerebellar nuclei. V, vestibular nuclei. Bar = 500 μm **(A,B)**, 200 μm **(D,F,G)**, 30 μm **(C)**, and 20 μm **(E,H,I)**.

The *tambaleante* mutation (*tbl*) is a recessive mutation that appeared spontaneously in a DW/J-Pas background at the Pasteur Institute (Paris, France). This mutation affects cerebellar Purkinje cells, leading to their disappearance (Wassef et al., 1987). In the homozygous *tambaleante* (*tbl/tbl*) mutation, the degeneration of Purkinje cells begins from the second month of life—a date from which the Purkinje cell number decreases dramatically. When the *tbl/tbl* mouse is 1 year old, fewer than 1% of the cerebellar Purkinje cells survive (Dusart et al., 2006) (Figure 2). The gene related to the mutation is *Herc1*, which expresses a protein involved in the growth and maintenance of the cerebellar cytoarchitecture. Moreover, there seems to be a relationship between the increased levels of the mutated protein HERC1 and the autophagic death of the Purkinje cells in the *tambaleante* mouse (Mashimo et al., 2009).

The *tambaleante* (*tbl/tbl*) mutation develops an ataxic syndrome (Wassef et al., 1987; Rossi et al., 1995), with signs of tremor, unstable gait, and abnormal posture of hind limbs, similar to that in other mutated cerebella devoid of Purkinje cells (Table 1). However, the phenotype of the *tbl/tbl* mouse and the regressive phenomena that will take place “en cascade” as in other Purkinje-cell-mutated cerebella (Sotelo and Changeux, 1974a; Caddy and Biscoe, 1979) are not completely understood. Thus, data are still not available as to whether the *tbl/tbl* mutation could affect only cerebellar Purkinje cells or whether other cerebellar and extracerebellar neuronal cell populations would also be affected directly or indirectly by the mutation. Despite the lack of concrete data on the extent of the *tbl/tbl* mutation, the late onset of complete Purkinje-cell degeneration with respect to other mutations (Table 1), once all cerebellar circuits have developed normally, makes it a perfect model for studying the cerebellar involvement in various motor learning tasks.

**Table 1:** **Main mutations affecting cerebellar Purkinje cells (Pc)**.

**Mutation**	**Onset of Pc degeneration**	**End of Pc degeneration**	**Size of Pc degeneration**	**Cerebellar and pre-cerebellar associated cell degenerations**	**Associate neural degenerative changes**	**Motor impairment**	**References**
Leaner (*tg*^1*a*^/tg^1*a*^)^1^	P15–40	5, 6 month	Alternate Pc bands with bands devoid of Pc^2^	Golgi neurons^3^, granule cells^3^, inferior olivary neurons^4^	Hippocampus^5, 6^	Most severe that in other mutations^6^	^1^Dickie (1962) ^2^Heckroth and Abbott (1994) ^3^Herrup and Wilczynski (1982) ^4^Zanjani et al. (2004) ^5^Alonso et al. (2008) ^6^Grüsser-Cornehls and Bäurle (2001)
Lurcher (*Lc*/+)^1^	P3–4	4 month	Complete	Inferior olivary neurons^2^, granule cells^2^	No data available	Mild^3^	^1^Phillips (1960) ^2^Caddy and Biscoe (1979) ^3^Grüsser-Cornehls and Bäurle (2001)
Nervous (*nr/nr*)^1^	P23	P50	Alternate Pc bands with bands devoid of Pc^2^	Inferior olivary neurons^3^	Retinal photoreceptors^4^	Mild^5^	^1^Sidman and Green (1970) ^2^Wassef et al. (1987) ^3^Zanjani et al. (2004) ^4^Mullen and LaVail (1975) ^5^Grüsser-Cornehls and Bäurle (2001)
Pogo (*pogo/pogo*)^1^	P120	–	Vermal Pc^2^	No data available	No data available	Mild^1, 2^	^1^Lee and Jeong (2009) ^2^Jeong et al. (2000)
Purkinje cell degeneration (*pcd/pcd*)^1^	P15	P45	Complete	Inferior olivary neurons^2^, granule cells^3^, cerebellar nuclei neurons^4^	Retinal photoreceptors^1^, olfactory bulb mitral cells^1^, thalamic neurons^5^	Mild^6^	^1^Mullen et al. (1976) ^2^Ghetti et al. (1987) ^3, 4^Triarhou et al. (1985); Triarhou et al. (1987) ^5^O'Gorman (1985) ^6^Grüsser-Cornehls and Bäurle (2001)
Reeler (*rl/rl*)^1^	P0	P15	≤50% with ectopic remaining Pc^2^	GABAergic interneurons^3^, granule cells^4^, unipolar brush cells^4^	Hippocampus^5^, neocortex^5^	Mild	^1^Falconer (1951) ^2^Heckroth et al. (1998) ^3^Takayama (1994) ^4^Ilijic et al. (2005) ^5^Park and Tom Curran (2008)
Staggerer (*sg/sg*)^1^	P0	P24	75% with ectopic remaining Pc^2^	All granule cells^2^, inferior olivary neurons^3^	Hippocampus^4^, olfactory bulb^5^	Most severe than in *Lc*, *nr*, or *pcd*^6^	^1^Sidman et al. (1962) ^2^Sotelo and Changeux (1974a) ^3^Zanjani et al. (2007) ^4^Yi et al. (2010) ^5^Deiss et al. (2001) ^6^Grüsser-Cornehls and Bäurle (2001)
Tambaleante (*tbl/tbl*)^1^	P60	4–6 month	Complete^1^	No data available	No data available	Mild, like *Lc* or *pcd*^2, 3^	^1^Wassef et al. (1987) ^2^Rossi et al. (1995) ^3^present observations
Weaver (*wv/wv*)^1^	P0	P14	25% with great alteration of the dendritic trees of remaining Pc^2, 3^	Granule cells^2, 3^	Hippocampus^4^, substantia nigra pars compacta^5^	Most severe than in *Lc*, *nr*, *pcd, or sg*^6^	^1^Lane (1965) ^2^Sotelo and Changeux (1974b) ^3^Sotelo (1980) ^4^Sekiguchi et al. (1995) ^5^Schmidt et al. (1982) ^6^Grüsser-Cornehls and Bäurle (2001)

Recent studies carried out in our lab show that the *tbl/tbl* mouse seems not to have its motor learning capabilities completely affected (Figure 1). Thus, although slower than wild-type mice, *tbl/tbl* mice perform both fall and horizontal bar test successfully (Figures 1A,B), learning consistently from the first to last session. However, *tbl/tbl* mice were unable to adapt their motor responses in the vertical pole test, in which they systematically failed to learn through the four sessions (Figure 1C).

Purkinje cell loss elicits a series of compensatory structural changes in the main cerebellar output (i.e., from deep cerebellar and vestibular nuclei). The diminution on Purkinje cell inhibitory input leads to changes in these neuronal populations that have been closely related to mutant behavioral phenotypes. Among these neural responses, an increase in the parvalbumin (Parv) was consistently found in cerebellar and vestibular nuclei after spontaneous or surgical Purkinje cell deprivation (Grüsser-Cornehls and Bäurle, 2001). In DW/J-Pas wild-type mice, deep cerebellar and vestibular nuclei neuronal somata are Parv- (Figure 2D), while small Parv+ terminal endings are present (Figure 2E). As in the *Lc*/+ mutation (Grüsser-Cornehls and Bäurle, 2001), Parv+ somata are found through all deep cerebellar nuclei in *tbl/tbl* mice (Figures 2F,G). Parv immunoreactivity of presynaptic boutons is also different in *tbl/tbl* deep cerebellar nuclei, and—together with small boutons (Figure 2I)—there is an increase in the presence of larger Parv+ endings (Figure 2H). Therefore, Parv immunoreactivity of *tbl/tbl* mice is similar to that observed in other Purkinje-cell-deprived mutations, reinforcing the relationships between motor behavior and Parv expression in deep cerebellar and vestibular nuclei (Grüsser-Cornehls and Bäurle, 2001).

Despite the similarities in the structural changes suffered by the different Purkinje-cell-devoid mutant mice sharing a similar motor behavior (Table 1), there are subtle differences in the motor response of the two strains analyzed here. Thus, while no differences were found in the fall test (Figure 1A), *tbl/tbl* mice seemed to perform the horizontal bar test more easily than did *Lc*/+ ones (Figure 1B). In contrast, *tbl/tbl* mice were consistently unable to successfully perform the vertical pole test, while *Lc*/+ mice did (Figure 1C). These differences in motor learning could be due to dissimilarities between the two mutant mice in the structural changes in cerebellar connectivity as the result of Purkinje cell loss. A possible explanation of these differences could reside in the different temporal onset of Purkinje-cell degeneration, as *tbl/tbl* is the only mutation that losses all Purkinje cells once the cerebellar circuits have developed normally (Wassef et al., 1987; Rossi et al., 1995; Dusart et al., 2006) (Table 1). A detailed analysis of the *tbl/tbl* cerebellum regarding the total amount of granule cells after Purkinje cell loss, and the cerebellar cortico-nuclear relationships at the beginning of the mutation effect, would explain these motor learning differences. Accordingly, the comparison of amounts of GABAergic input to cerebellar deep nuclei between *Lc*/+ and *tbl/tbl* mice could help to solve the question (see Grüsser-Cornehls and Bäurle, 2001). However, it is noteworthy that genes leading to Purkinje cell degeneration also affect other brain regions, and that ataxic symptoms and motor behavior are most severe in mutant mice whose brain is widely affected (Table 1). Hence, the analysis of *tbl/tbl* brain areas involved in motor behavior and the possible compensatory processes taking place after the loss of Purkinje cells when all motor circuitry is fully developed would explain the differences in *tbl/tbl* motor behavior.

## Conclusion

Various studies give the cerebellar cortex an important role in motor learning. However, the results obtained in our laboratory, using two Purkinje-cell-deprived mutant mice, show that while this structure is not essential in learning, its absence disturbs the performance and the magnitude of the learned response (Porras-García et al., 2005, 2010). This is not so in the case of the interpositus nucleus of the cerebellum, in which any injury causes a total lack of learning (Porras-García et al., 2010). In accord with our results and those of some other authors, it seems motor learning must be due to coordinated work between several cerebellar and extra-cerebellar structures.

### Conflict of interest statement

The authors declare that the research was conducted in the absence of any commercial or financial relationships that could be construed as a potential conflict of interest.
